# Inhibition of Asthma in OVA Sensitized Mice Model by a Traditional Uygur Herb* Nepeta bracteata* Benth.

**DOI:** 10.1155/2016/5769897

**Published:** 2016-03-17

**Authors:** Jing Wang, Feng-sen Li, Nan-nan Pang, Ge Tian, Min Jiang, Hong-ping Zhang, Jian-bing Ding

**Affiliations:** ^1^Xinjiang National Clinical Research Base of Traditional Chinese Medicine, Xinjiang Medical University, Urumqi, Xinjiang 830000, China; ^2^College of Basic Medicine, Xinjiang Medical University, Urumqi, Xinjiang 830011, China; ^3^First Affiliated Hospital of Xinjiang Medical University, Urumqi, Xinjiang 830011, China

## Abstract

Asthma is a chronic lung inflammation which affects many people. As current therapies for asthma mainly rely on administration of glucocorticoids and have many side effects, new therapy is needed. In this study, we investigated* Nepeta bracteata* Benth., a traditional Uygur Herb, for its therapeutics effect in OVA induced asthmatic mice model. Treatment of OVA sensitized asthma mice with extract from* Nepeta bracteata* Benth. demonstrated improved lung pathology, as well as reduced infiltration of eosinophil and neutrophil.* Nepeta bracteata* Benth. extract also contributed to the rebalance of Th17/Treg cell via decreasing the Th17 cell and increasing the Treg, which was corresponding with the inhibited Th17 cytokine response and increased IL-10 level. Moreover, the reduced TGF-*β* level and Smad2/3 protein level also suggested that* Nepeta bracteata* Benth. extract could inhibit TGF-*β* mediated airway remodelling as well. Taken together, these data suggested that* Nepeta bracteata* Benth. may be a novel candidate for future antiasthma drug development.

## 1. Introduction

Asthma is a chronic inflammatory disease of the airways and is characterized by variable and recurring symptoms, as well as airflow obstruction and bronchospasm. Common symptoms of asthma include wheezing, coughing, chest tightness, and shortness of breath. According to the report of World Health Organization, nearly 300 million people were newly diagnosed with asthma annually and about 250.000 deaths were associated with this disease.

Traditional treatments for asthma include inhaled corticosteroids (ICS), short-acting *β*2-agonist (SABA), leukotriene receptor antagonists (LTRAs), and long-acting *β*2-agonist (LABA) [1, 2]. However, these treatments still failed in some nonresponsive patients. It is believed that the nonresponsiveness to these treatments is caused by some inflammatory processes that are not targeted by currently therapies [2]. Moreover, in recent years, as the role of Th17 cell had been explored for the initiation and progression of asthma, monoclonal antibody targeted human IL-17 receptor had been examined in clinical trials for asthma patients [2].

Except the treatments mentioned above, herbs based traditional medicine had been used for asthma treatment as well. Studies from Japan had demonstrated that Saiboku-To, a herbal extract mixture which came from traditional Chinese medicine, was able to relieve the asthma through inhibiting type I hypersensitivity reaction via the suppression of histamine release [3, 4]. Boswellia, another herb used in Ayurvedic medicine (a traditional Indian system of health care), had been shown to relieve the asthma in patients with different ages in clinical trial [5]. Pycnogenol, a standardized extract from French maritime pine bark, suggested that it could reduce the symptoms in asthma patients and improve their lung function [6–8].

In this study, the effects of a traditional Uygur Herb,* Nepeta bracteata* Benth., had been investigated for its therapeutics effect on OVA induced asthmatic mice model. Treatment of mice with* Nepeta bracteata* Benth. extract demonstrated improved pathology of the lung. Further analysis demonstrated that the infiltration of eosinophil and neutrophil was reduced in treated asthma mice.* Nepeta bracteata* Benth. extraction administration also contributed to the rebalance of Th17/Treg cell via decreasing the Th17 cell. On the other hand, the decreased cytokines level indicated that Th17 mediated response was inhibited as well. Moreover, the reduced TGF-*β* level and Smad2/3 protein level also suggested that* Nepeta bracteata* Benth. extract could inhibit TGF-*β* mediated airway remodelling in mice. Taken together, those data indicated that* Nepeta bracteata* Benth. could be a good candidate for novel antiasthma drug development.

## 2. Materials and Methods

### 2.1. Ethics Statement

The animal protocols used in this study were approved by the Animal Disease Prevention and Control Center and followed the instruction of the* Guidelines for Experimental Animals* which is issued by the Ministry of Science and Technology (Beijing, China). All procedures were performed according to recommendations proposed by the Animal Disease Prevention and Control Center, and all efforts were made to minimize suffering of mice. Moreover, the mice were housed in a temperature-controlled room with proper darkness-light cycles, fed with a regular diet, and maintained under the care of the Experimental Animal Center.

### 2.2. Preparation of Herbal Extract

After 2000 g crude* Nepeta bracteata* Benth. herb was smashed and the crude was repeatedly dissolved in distilled water for 3 times. The solvents were extracted via rotary evaporation under reduced pressure. Then the 95% ethanol was added to the remaining extract with a v/v ratio at 4 for overnight precipitation. After precipitation, the savage agent was used to remove the protein carryover. Then the extract was lyophilized and aqueous extract was prepared by dilution with PBS before use.

### 2.3. Animal and Asthma Mice Model

12-week-old BALB/c mice (*n* = 70, female) were randomly divided into six groups: control, asthma group, low dose group, medium doses group, high dose group, and dexamethasone group. The OVA sensitization of mice was conducted as previously described [9]. 15 days after OVA sensitization, 5% OVA was atomized and inhaled by mice daily to induce asthma. For control group, PBS was used instead of the OVA. The herbal extract and dexamethasone were administrated daily via intragastric tube. The dose used for different groups was 0.5 mg/kg for dexamethasone and 1.2 g/kg, 0.9 g/kg, and 0.6 g/kg for high dose, medium dose, and low dose groups of herbal extract, respectively.

### 2.4. Histology Analysis

24 hours after the last OVA challenge, the mice were euthanized for specimen collection. The left lung was removed and fixed in 4% formaldehyde. Slides with paraffin-embedded sections were stained by hematoxylin-eosin staining for pathological analysis.

### 2.5. Bronchoalveolar Lavage Fluid Cell Counting

The collection of bronchoalveolar lavage fluid (BALF) was previously described [10]. The BALF samples were prepared by microscope slide smear and cytocentrifugation. The total cell number, macrophage, lymphocyte, eosinophils, and neutrophils were counted by using Wright's stain and the differentials were compared between different groups of mice.

### 2.6. Flow Cytometry Analysis

Total lymphocytes from mouse peripheral blood and spleens were isolated by Ficoll Paque Plus (Sigma-Aldrich) according to the manufacturer's instruction. Th17 and Treg cell were stained by Mouse Th17/Treg phenotyping kit CD4 (PerCP-Cy5.5), IL-17 (PE), and foxP3 (Alexa Fluor® 647) (BD Bioscience) by following manufacturer's instruction.

### 2.7. Reverse Transcription and Qualitative Real-Time PCR (qPCR)

Total RNA was extracted from the mice lung samples by using TRIzol® Reagent (Invitrogen) according to manufacturer's instruction. RNase-free DNase treatment was conducted to remove genome DNA contamination. For the detection of indicated gene expression in RNA level, reverse transcription was carried out by using AMV reverse transcriptase (Promega) and a combination of random primer and oligo(dT) as previously reported [11]. Real-time PCR with SYBR Green detection was done as described previously [12]. Transcripts of house-keeping gene RPL32 (ribosomal protein L32) were also amplified from the same samples to serve as an internal control for cellular mRNA normalization. Expression of indicated genes was quantified by 2^−ΔΔCT^ method as previously described [13]. The primers were designed as follows: IL-6 F: GAC­TGA­TGC­TGG­TGA­CAA­CC R: CTC­TCT­GAA­GGA­CTC­TGG­CTT, IL-10 F: TGC­CTG­CTC­TTA­CTG­ACT­GG R: AAT­GCT­CCT­TGA­TTT­CTG­GGC, IL-17A F: TGT­GTC­TCT­GAT­GCT­GTT­GCT R: AAC­GGT­TGA­GGT­AGT­CTG­AGG, TGF-*β* F: CAA­ACT­AAG­GCT­CGC­CAG­TC R: TGC­TTC­CCG­AAT­GTC­TGA­CG, Smad2 F: TAT­CAC­TGC­TTC­CCT­TCC­GC R: TGA­CTT­GTT­CAC­GCT­CGG­T, Smad3 F: TAG­GAG­TAA­AGG­GAG­CGG­GT R: AAG­GAG­TCA­GGT­GGC­GAT­AC, *β*-actin F: CAG­GGT­GTG­ATG­GTG­GGA­AT R: GTA­GAA­GGT­GTG­GTG­CCA­GAT.

### 2.8. Western Blot Analysis

Total protein was isolated from the right lung by homogenization with a buffer as previously described [14]. The samples were clarified by centrifugation at 14000 g for 5 min to remove the debris and intact cell. Then the supernatant was lysed in Laemmli sample buffer. The whole proteins in the lysate were analyzed by sodium dodecyl sulfate-polyacrylamide gel electrophoresis (SDS-PAGE) and Western blot as described previously [15, 16]. Antibodies against Smad2/3 (Santa Cruz Biotechnology, Santa Cruz, CA), Smad7 (Santa Cruz Biotechnology), and actin (Sigma-Aldrich, St. Louis, MO) were used in the blotting. The chemiluminescence signal was recorded digitally using a ChemiDoc XRS imaging system (Bio-Rad Laboratories, Hercules, CA). Digital signal acquisition and densitometry analyses were conducted using the Quantity One Program, Version 4.6 (Bio-Rad).

### 2.9. ELISA Analysis for Cytokine Expression

Mice serum samples were collected as previously described [17]. ELISA kits used for the measurement of cytokine level (IL-4, IL-6, IL-10, IL-17a, and TGF-*β*) were obtained from R&D system (R&D Systems, Minneapolis, MN). ELISA procedures were conducted by following manufacturer's instructions.

### 2.10. Statistical Analysis

Differences in indicators between treatment samples, such as cytokines level between the different mice groups, were assessed by Student's *t*-test. A two-tailed *P* value of less than 0.05 was considered significant.

## 3. Results

### 3.1. Histology Examination Demonstrated Reduction of Pathologic Change in Lung Tissue of Mice Given* Nepeta bracteata* Benth. Extract 

**Figure 1:**
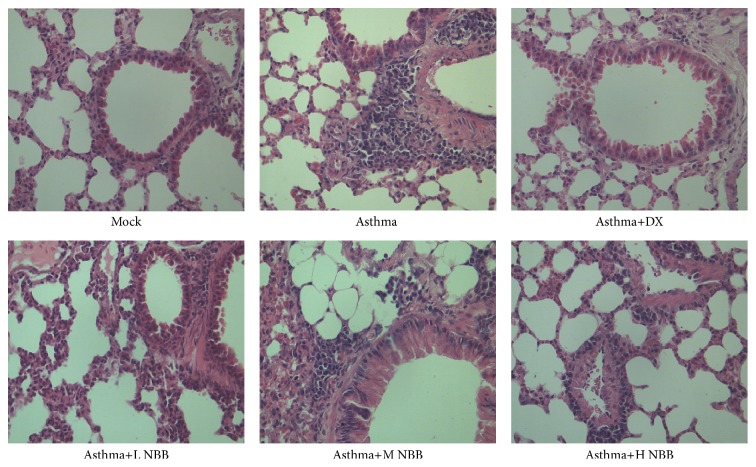
Histology examination demonstrated a reduction of pathologic change in lung tissue of mice given* Nepeta bracteata* Benth. extract.

Historically,* Nepeta bracteata* Benth. had been used as traditional medicine for the asthma treatment in Xinjiang. However, the detailed research focusing on mechanism of this herb has not been conducted yet. To confirm the therapeutic effect of* Nepeta bracteata* Benth. for asthma, we made crude extract of this herb as described in Section 2 and set 3 different dose groups: low, medium, and high. An OVA induced mice asthma model had been used in this study. In our experiment, induction of asthma via OVA in mice resulted in significant morphology changes in the lung tissue, such as infiltration of proinflammatory lymphocytes and damage in airway epithelial cell (Figure 1). However, after administration of* Nepeta bracteata* Benth. extract, there was improvement of morphology in asthma mice, which indicated that this herb extract could reduce the symptoms of asthma. The medium dose group specially demonstrated the best improvement among all three dose groups (Figure 1). On the other hand, we also examined different cell types in bronchoalveolar lavage fluid. The medium dose group showed the reduced infiltration of lymphocyte, eosinophil, neutrophil, and macrophage among all three dose groups, which suggested reduced inflammation response in mice given herb extract (Table 1). Taken together, these findings confirmed the therapeutic effect of* Nepeta bracteata* Benth. on asthma.

### 3.2. *Nepeta bracteata* Benth. Extract Administration Could Reduce the Th17 Cell and Rebalance of Th17/Treg

**Figure 2:**
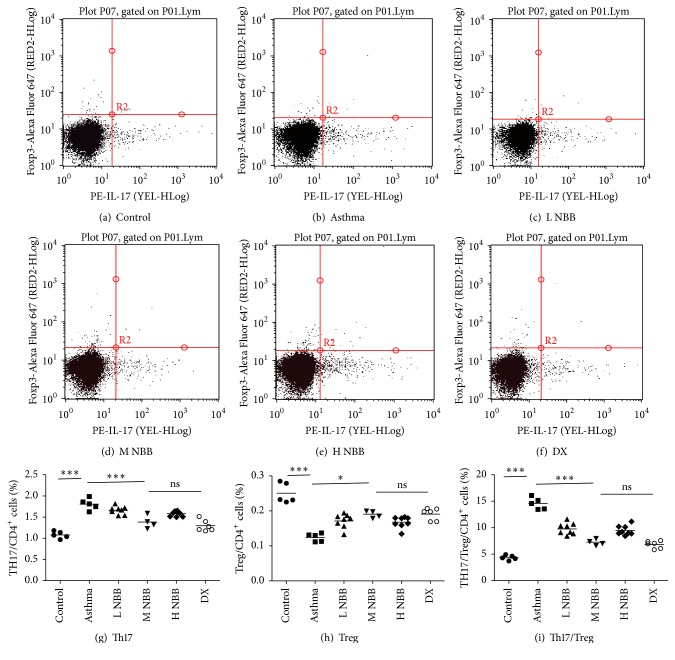
Frequencies of Th17 and Treg cells, and the ratio of Th17 to Treg in the peripheral blood mononuclear cells by flow cytometry (the proportion of Th17 cells (g) and Treg cells (h) among CD4^+^ cells and the ratio of Th17 to Treg (i) were shown as scatter plot graphs. ^*∗∗∗*^ *P* < 0.001; ^*∗*^ *P* < 0.05, ^ns^ *P* > 0.05).

Recently, the roles of Th17 cell played during the progression of asthma had been exclusively investigated. Although our data suggested that* Nepeta bracteata* Benth. extract could relieve the symptoms of asthma in mice model, the mechanism is still unknown. We next tested whether* Nepeta bracteata* Benth. extract could reduce the number of Th17 cells and contribute the rebalance of Th17/Treg ratio. In flow cytometry analysis, the medium dose group still demonstrated the strongest reduction of Th17 cell among three dose groups and reached a comparable level to the dexamethasone treated mice (Figure 2(g)). On the other hand, the number of Treg cells was also increased in the low dose and medium dose groups and contributed to the rebalance of Th17/Treg ratio (Figures 2(h) and 2(i)). However, in the high dose group, the total Treg cell number was unexpectedly dropped to the lowest among all herbal extract treated and dexamethasone treated groups (Figure 2(i)). This may explain why application of high dose extract in mice did not improve the lung morphology in histological analysis. However, the reason that led to this observation was unknown, maybe due to the toxicity generated by high dose extract.

### 3.3. *Nepeta bracteata* Benth. Extract Reduced the Th17 Mediated Proinflammatory Cytokine Expression in Mice

**Figure 3:**
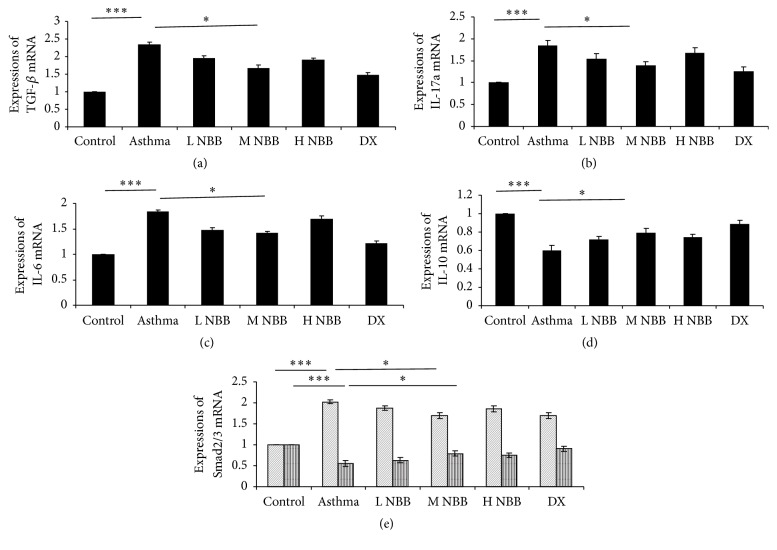
Expressions of (a) TGF-*β*, (b) IL-17a, (d) IL-10, (c) IL-6, and (e) Smad2/3 mRNA in different groups (^*∗∗∗*^ *P* < 0.001; ^*∗*^ *P* < 0.05).

**Figure 4:**
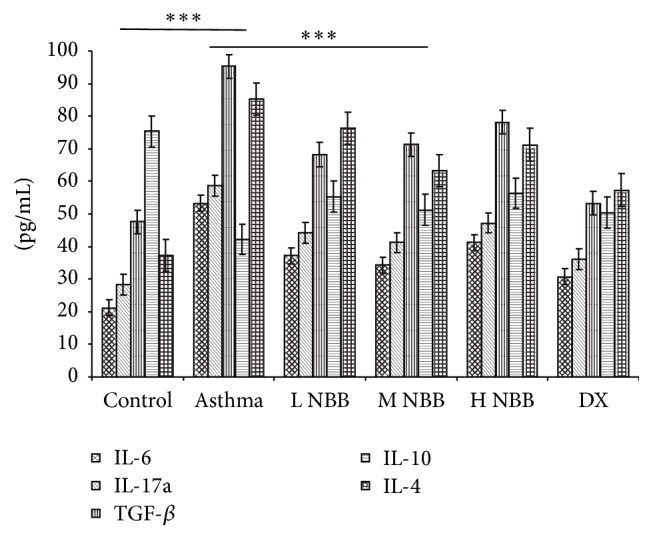
Expressions of TGF-*β*, IL-17a, IL-10, IL-6, and IL-4 proteins in the peripheral blood (^*∗∗∗*^ *P* < 0.001).

As* Nepeta bracteata* Benth. extract administration reduced the Th17 cell and contributed to the rebalance of Th17/Treg, we next checked the cytokines expression in the extract treated mice. As we expected, the mRNA level of IL-6 and IL-17a is dropped in the medium dose group (Figure 3) and was consistent with the ELISA results for them (Figure 4). These data suggested that Th17 mediated proinflammatory response was inhibited by* Nepeta bracteata* Benth. extract treatment. On the other hand, the IL-10 was also evaluated both in mRNA level and in protein level in medium dose extract treated mice as a consequence of rebalance of Th17/Treg (Figures 3 and 4), which was consistent with our observation in flow cytometry analysis as well. Moreover, we also examined the TGF-*β* mRNA level and protein level via qPCR and ELISA. The reduced TGF-*β* may also contribute to the therapeutic effect of* Nepeta bracteata* Benth. extract to asthma (Figures 3 and 4).

### 3.4. *Nepeta bracteata* Benth. Extract Modulates Expression of Downstream Molecule of TGF-*β*

**Figure 5:**
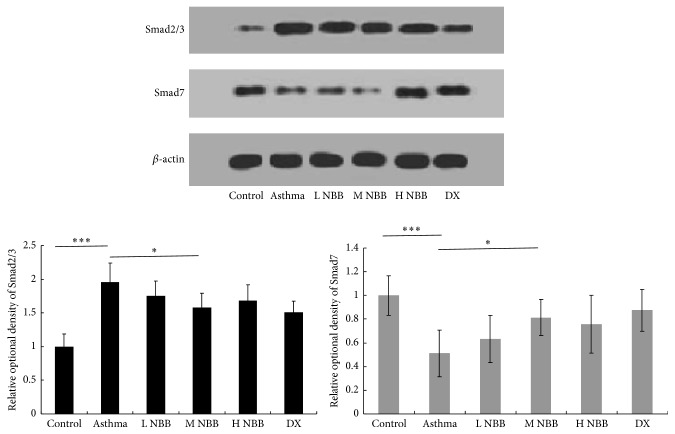
*Nepeta bracteata* Benth. extract modulates the expression of downstream molecule of TGF-*β* (^*∗∗∗*^ *P* < 0.001; ^*∗*^ *P* < 0.05).

Since our data demonstrated that TGF-*β* level is reduced in mice groups given* Nepeta bracteata* Benth. extract, we would like to know whether the extract could affect the TGF-*β* signaling as well (Figure 5). After the binding of TGF-*β* to its receptor, the downstream signaling is transduced by two transcription factors Smad2 and Smad3 and the signaling could be modulated by another Smad family member Smad7 [14]. For the expression of Smad2/3, the medium dose administration of* Nepeta bracteata* Benth. extract appears to demonstrate the strongest inhibition. However, the inhibition level is still much lower compared with dexamethasone treated group.

## 4. Discussion

For a long time, the treatment of asthma relies on application of glucocorticoids. However, besides the side effect of glucocorticoids, some patients were nonresponsive for glucocorticoids. On the other hand, as human monoclonal antibodies targeting specific cytokine receptors based therapy are under clinical trials, the antigenicity for these antibodies in human needs to be considered. Therefore, new treatment based on herbs which came from traditional medicine provides a novel way for fighting asthma. In this study, Uygur Herb,* Nepeta bracteata* Benth., had been investigated for its therapeutics effect in OVA induced asthma in mice model. Our data suggested that* Nepeta bracteata* Benth. extract could function in multiple levels against asthma.

Th17 cell could secrete the IL-17a, IL-17f, and IL-6 and TNF-*α*; these cytokines play important roles in activating neutrophils, which contributes to the inflammatory process [18]. In the development of asthma, not only did Th17 enhance the Th2-cell-mediated eosinophilic airway inflammation [19], but also data indicated that IL-17 mRNA level was correlated with infiltration of neutrophil and severity of asthma [19, 20]. In our study, our result suggested that* Nepeta bracteata* Benth. extract could reduce the Th17 cell number when administrated to mice. This observation is also consistent with reduced infiltration of eosinophils and neutrophils in bronchoalveolar lavage fluid cell counting, which suggested that Th17 mediated response is suppressed by application of* Nepeta bracteata* Benth. extract. On the other hand, ELISA results also demonstrated that expression of IL-4, IL-6, and IL-17a was reduced when compared with asthma induced mice and further confirmed that the inflammation response could be inhibited by* Nepeta bracteata* Benth. extract.

As the role of Treg had been explored in the development of asthma, it had been demonstrated that Treg could reduce OVA induced allergic airway inflammation in mice model [21]. Further research also indicated that the imbalance of Th17/Treg contributed to asthma progress and rebalance of Th17/Treg could be a potential therapeutic target [22, 23]. Our data also suggested that administration of* Nepeta bracteata* Benth. extract could increase the Treg number in blood and somehow rebalance the ratio of Th17/Treg to a similar level of dexamethasone treated group, which may contribute to its therapeutic effect of asthma. However, neither medium dose herbal extract group nor dexamethasone group could achieve similar Th17/Treg ratio as control group, which suggested that the monotherapy of dexamethasone or herbal extract may not be effective enough to completely rebalance the Th17/Treg. It is possible that a combination of glucocorticoid therapy and herbal extract which came from traditional medicine could be more superior than monotherapy.

Interestingly, although we set different dose for the* Nepeta bracteata* Benth. extract administration to mice, it appears that the medium dose had the best performance for asthma treatment. Since we still used the crude extract in this study and the exact component of the* Nepeta bracteata* Benth. extract contributing to the asthma therapy is still unknown and the crude extract may contain other substances as impurities, further investigation to identify and purify the active ingredient of the extract is needed. It is also possible that high dose administration of crude extract also introduced more impurities than the low dose to mice, which may cause side effect on asthma treatment.

Moreover, except the benefit mentioned above, our data also suggested that* Nepeta bracteata* Benth. extract could inhibit the TGF-*β* expression in both mRNA level and protein level. Transforming growth factor-b1 (TGF-*β*) is a profibrotic cytokine which had been considered to promote the structural changes of airway remodelling during the asthma [14]. After the binding of TGF-*β* to its receptors, two nuclear factors Smad2/3 will translocate to the nucleus and activate downstream genes transcription [14]. Our data suggest that asthma induced Smad2/3 expression is inhibited by* Nepeta bracteata* Benth. extract, which could dampen the TGF-*β* signaling. On the other hand, as the Smad7 is an intracellular inhibitor to provide the negative feedback for TGF-*β* signaling, it seems that the Smad7 expression has no change in* Nepeta bracteata* Benth. extract treated mice when compared with asthma mice (data not shown), which suggests that Smad7 expression is not targeted by extract for inhibiting TGF-*β* signaling. Taken together, our data provide the evidences for the therapeutic effect of* Nepeta bracteata* Benth. extract for asthma and this herb will be a good candidate for the future asthma treatment.

## Figures and Tables

**Table 1:** The percentage change of different cell type in bronchoalveolar lavage fluid.

Group	*N*	Total cells (10^4^/mL)	Differential leukocyte count (%)			
			Lymphocyte	Eosinophil	Neutrophil	Macrophage
Control	6	15.3 ± 1.5	36.6 ± 2.5	1.2 ± 0.3	7.7 ± 1.1	54.5 ± 3.3
Model	6	137.7 ± 11.2	53.3 ± 1.4	10.9 ± 0.5	23.1 ± 1.2	12.7 ± 0.5
Low	6	105.2 ± 9.3	50.2 ± 1.5	9.2 ± 0.6	19.6 ± 1.5	21 ± 1.9
Medium	6	81.4 ± 6.4	45.9 ± 1.8	7.8 ± 0.4	16.8 ± 1.0	29.5 ± 2.1
High	6	97.7 ± 7.5	44.7 ± 1.2	7.6 ± 1.1	20.5 ± 1.1	27.2 ± 0.9
Positive	6	62.7 ± 8.6	38.7 ± 2.1	5.3 ± 0.3	13.8 ± 1.2	42.2 ± 1.8
